# Microbial Indicators of Fecal Pollution: Recent Progress and Challenges in Assessing Water Quality

**DOI:** 10.1007/s40572-020-00278-1

**Published:** 2020-06-15

**Authors:** David A. Holcomb, Jill R. Stewart

**Affiliations:** 1 Department of Epidemiology, Gillings School of Global Public Health, University of North Carolina at Chapel Hill, 135 Dauer Dr., Chapel Hill, NC 27599-7435 USA; 2 Department of Environmental Sciences and Engineering, Gillings School of Global Public Health, University of North Carolina at Chapel Hill, 135 Dauer Dr., Chapel Hill, NC 27599-7431 USA

**Keywords:** *Escherichia coli*, Environmental antimicrobial resistance, Fecal indicator bacteria, Microbial source tracking, qPCR, Water quality

## Abstract

**Purpose of Review:**

Fecal contamination of water is a major public health concern. This review summarizes recent developments and advancements in water quality indicators of fecal contamination.

**Recent Findings:**

This review highlights a number of trends. First, fecal indicators continue to be a valuable tool to assess water quality and have expanded to include indicators able to detect sources of fecal contamination in water. Second, molecular methods, particularly PCR-based methods, have advanced considerably in their selected targets and rigor, but have added complexity that may prohibit adoption for routine monitoring activities at this time. Third, risk modeling is beginning to better connect indicators and human health risks, with the accuracy of assessments currently tied to the timing and conditions where risk is measured.

**Summary:**

Research has advanced although challenges remain for the effective use of both traditional and alternative fecal indicators for risk characterization, source attribution and apportionment, and impact evaluation.

## Introduction

Fecal contamination of water continues to be a major public health concern, with new challenges necessitating a renewed urgency in developing rapid and reliable methods to detect contamination and prevent human exposures. Aging sewer infrastructure in the USA and elsewhere will require rapid methods to assess fecal contamination of water [1, 2•, 3]. The number of extreme weather events including flooding events is forecast to increase with climate change and has been associated with contamination of water resources [4–6]. Also, the increasing threat of antimicrobial resistance is making it all the more important to lower the rates of infections across the globe, especially infections that require antibiotic treatment, and to identify environments contaminated with antibiotic-resistant pathogens [7–9].

Fecal indicator bacteria have been used for over 150 years to indicate fecal contamination of water and associated health risks (Table 1). The latter half of the nineteenth century saw the discovery of waterborne disease transmission, perhaps most famously in the analysis of drinking water systems by John Snow during the 1854 London cholera outbreak and the isolation of *Vibrio cholerae* by Robert Koch in 1884 (though first identified in 1854 by Filippo Pacini) [10–12]. Recognition that sewage contamination of water sources spreads diseases such as cholera and typhoid necessitated a means by which to ascertain the presence of sewage in drinking water. Coliform bacteria, a group of typically harmless Gram-negative bacteria that constitute part of the natural gut microbiota in humans and other warm-blooded animals, provided a simple and reasonably reliable tool for diagnosing sewage pollution in drinking water samples owing to their high concentrations in sewage and ease of culture [13, 14]. A growing concern about fecal pollution in the wider environment and the potential for human exposure to enteric pathogens through additional environmental pathways, especially recreational and foodborne exposure routes, encouraged subsequent efforts to assess fecal contamination in an increasing variety of environmental matrices [15, 16]. Feces can contain a wide range of pathogens, which when introduced to the environment may persist for varying amounts of time, often at concentrations too low for reliable detection but still hazardous to human health [17–19]. Furthermore, conventional methods of enteric pathogen detection are generally time-consuming, expensive, and often insensitive even in fresh feces [20]. The use of fecal indicator bacteria (FIB) like the fecal coliform *Escherichia coli* to suggest the presence of hazardous fecal pollution therefore continues to be a valuable tool to assess water quality.

**Table 1:** Indicators of fecal contamination for water quality assessments

Indicator	Example targets	Applications	Stage of development
Fecal indicator bacteria—culture-based detection	*E. coli*, enterococci	Hazard identification, regulatory compliance	Late
Fecal indicator bacteria—molecular detection	*E. coli*, enterococci	Hazard identification, regulatory compliance	Middle
Fecal indicator viruses	Coliphages, *Bacteroides* bacteriophages	Assess risk from enteric viruses	Late
Human-associated MST markers	*Bacteroides* HF183, HumM2, PMMoV, crAssphage	Determine source of contamination	Middle
Animal-associated MST markers	BacCow, BacCan, avian GFD, Pig-2-Bac	Determine source of contamination	Middle
Index pathogens	Noroviruses, rotaviruses, *Salmonella* spp., *Campylobacter* spp., *Cryptosporidium* spp.	Risk assessment	Middle
Antimicrobial-resistant (AMR) bacteria	ESBL *E. coli*, MAR E. coli, MRSA	Assess environmental antimicrobial resistance	Early
Antimicrobial resistance genes (ARGs)	*int*I, *mcr-1*, *tnpA*, *sul*1, *tet*W, *tet*M, *qepA*, *bla*_TEM_	Assess environmental antimicrobial resistance	Early

*PMMoV* pepper mild mottle virus, *ESBL E. coli* extended-spectrum β-lactamase-producing *E. coli*, *MAR E. coli* multiple antibiotic–resistant *E. coli*, *MRSA* methicillin-resistant *Staphylococcus aureus*

Limitations of the fecal indicator paradigm have long been acknowledged [21–23]. Researchers have identified many challenges and limitations to the effective use of both traditional and alternative fecal indicators to characterize risk, identify sources, and evaluate interventions [24–26]. Arguably, one of the most significant limitations is the inconsistent relationships between FIB occurrence, enteric pathogens, and health risks [25, 27]. In settings with high rates of enteric infection and inadequate fecal waste management, *E. coli* in drinking water (but not other coliforms) has often been associated with increased risk of illness [28–30]. Similarly, the health risks of recreational uses of surface waters have been found to increase with FIB density, but generally only at locations with known human fecal inputs or under high-risk conditions, such as following precipitation or the removal of physical barriers [25, 31–35]. The FIB found to correlate with health risks also vary widely by site [32]. The co-occurrence of enteric pathogens and FIB in ambient waters is inconsistent at best [27, 36], and commonly used FIB are known to persist and grow in the environment [37–42].

In this paper, we review recent progress in the quest for improved indicators of fecal contamination in water. We summarize recent advances in alternative indicators with a focus on microbial source tracking markers. We also recognize the advances in molecular methods that are increasingly being used to detect fecal contamination of water and to identify sources of contamination. Improvements in detection capabilities, analytical sensitivity, and data quality are discussed along with barriers that must be overcome for wider adoption. We review efforts to characterize health hazards associated with fecal contamination, and we distinguish the timing and conditions when indicators appear best suited to identify risks to human health. Finally, we identify opportunities for continued improvements in the use of indicator organisms to assess environmental fecal pollution and to safeguard human health.

## Alternative Indicators

Many alternative fecal microbes have been proposed to address the limitations of traditional FIB as indicators of fecal pollution [21, 22, 43]. Better surrogates that share environmental fate and transport mechanisms with pathogens of concern—particularly coliphages, viruses that infect *E. coli* bacteria, and obligate anaerobes, thought to have host specificity and to derive exclusively from recent fecal contamination—have frequently been identified as potential alternative indicators expected to better represent risks to human health [44–46]. Health risks from exposure to ambient fecal contamination are largely a function of the specific pathogens present and their concentrations in the exposure matrix, which is strongly influenced by the source of the fecal pollution [47–51]. Enteric viruses that primarily derive from human sources, notably human noroviruses, drive the global burden of gastrointestinal illness, including from recreational water exposures [2•, 52–56]. Highly persistent and infectious protozoan parasites are shed at high rates by livestock [2•, 57–59], and avian sources introduce bacterial pathogens like *Campylobacter* spp*.* and non-typhoidal *Salmonella* spp., particularly the poultry common in domestic environments in low- and middle-income countries (LMIC) where frequent exposure is likely [57, 60–64]. Given the differences in human health risks and appropriate mitigation strategies for fecal pollution from different sources, there is a need to identify not only the presence of fecal contamination but also its origins. Because traditional FIB cannot discriminate between fecal sources, host-associated fecal microbes have been the subject of extensive research in recent years for use as indicators of source-attributable fecal pollution, an approach known as microbial source tracking (MST) [23, 43].

### Microbial Source Tracking

Numerous host-associated organisms and gene markers have been identified for identifying sources of fecal pollution in water, none of which has demonstrated perfect source sensitivity and specificity [46]. Numerous MST markers target members of the order *Bacteroidales*, many of which are obligate anaerobes and abundant constituents of the gut microbiota in warm-blooded animals, including one of the most common and earliest-proposed human-associated molecular markers, HF183 [43, 46, 65, 66]. Because MST targets specific constituents of the gut microbiota, the diagnostic performance of each MST marker can vary substantially between populations. Validation of existing MST markers for use in new geographic locations is increasingly standard practice [24], with recently published MST validation studies conducted in Australia [67, 68], Bangladesh [69, 70], Costa Rica [71], India [72], Japan [73, 74], Mozambique [75], New Zealand [76], Nepal [77, 78], Singapore [79], Thailand [80], and the USA [81, 82], and a global evaluation of markers in sewage from 13 countries on 6 continents [83•]. Potential MST markers continue to be identified, most notably human-associated crAssphage, a bacteriophage infecting *Bacteroides intestinalis* recently discovered to be an abundant, globally distributed constituent of the human gut virome [84–89]. Human-associated *E. coli* markers, long-desired for their direct correspondence to a common FIB used for regulatory purposes, have also been developed [90–92], though they may lack the analytical sensitivity for effective use in ambient waters [93]. The identification of new markers is increasingly supported by advances in sequencing technology and bioinformatics [84, 94–96], and next-generation sequencing (NGS)–based MST approaches continue to be refined [97]. Although highly dependent on fecal library composition (the collection of metagenomic sequences from known fecal sources that informs source identification algorithms) [97–101], NGS-MST has the potential to identify finer distinctions between sources, as demonstrated by a study in Kenya that distinguished between fecal contamination from young children and adults [102•]. The recent introduction of more affordable and portable long-read sequencing platforms, while currently error prone, promises to accelerate the use of sequencing to characterize fecal contamination [103, 104].

MST proponents typically advocate a “toolbox approach” to fecal source attribution that combines multiple MST markers, detection methods, and sampling strategies in recognition of the limitations of any single MST marker to reliably and conclusively characterize fecal pollution [105–107]. Two toolbox constituents recently receiving much attention in the literature are pepper mild mottle virus (PMMoV), a plant virus infecting *Capsicum* species acquired by humans from dietary sources, and crAssphage, both viruses that hold promise as human-associated viral surrogates owing to their global distribution in sewage at densities typically much higher than other viruses [2•, 71, 74, 78, 108–110]. Nonetheless, *Bacteroides* HF183 and its variants have arguably consolidated their role as the default tool for human source tracking [43], featuring consistently high concentrations in sewage globally [83•], frequent detection in surface waters [61, 93, 110–112], standardized protocols [81, 113], and validated multiplex assays [89, 114]. However, the diagnostic performance of HF183 and most other human-associated markers has typically been poor in highly contaminated settings in many low- and middle-income countries (LMIC) [58, 69, 70, 72, 75, 115], with the exception of high sensitivity to child feces in urban Kenya [102•].

Successful identification of non-human fecal sources may best demonstrate the value of MST for informing management and research priorities. Unlike human-associated markers, animal fecal markers (e.g., livestock-associated BacCow and canine-associated BacCan, both with *Bacteroidales* targets, and avian-associated GFD, which targets the genus *Helicobacter*) have performed well in LMIC settings and have repeatedly identified livestock as major sources of fecal contamination and pathogens in the domestic environment, supporting recent calls for renewed emphasis on animal waste management [58, 116]. Likewise, MST investigations can impact management and mitigation programs by determining that wildlife, livestock, or pets contributed substantially to fecal pollution in certain watersheds and beaches [81, 117•, 118•, 119]. The forensic potential of MST was demonstrated during a 2019 *Campylobacter* outbreak in Norway, which was attributed to non-human sources, most likely horses, using a combination of FIB, MST, and direct pathogen detection [120]. MST has also been used to identify sources of antimicrobial resistance, the environmental dimensions of which remain poorly understood [121, 122]. Similar investigations are likely to increasingly carry legal implications, for instance, by implicating animal agriculture industries in unpermitted surface water pollution [13, 123, 124].

## Molecular Methods: Challenges and Advances

The historic infeasibility of comprehensive direct pathogen detection in environmental waters continues to motivate the use of fecal indicators, which have traditionally been detected using culture-based methods. Growing FIB from water samples on selective media is routine and relatively inexpensive but generally requires a minimum of 18 hours to obtain results, by which time conditions at the sampling location may have dramatically changed [125–127]. Furthermore, the obligate anaerobes and host-specific viruses proposed as alternative indicators for MST are often not amenable to laboratory culture [43]. A range of alternative detection methods continue to be developed and are the subject of several recent comprehensive reviews [97, 128–131], with real-time polymerase chain reaction (qPCR) and related molecular methods that infer the presence of fecal microbes from their genetic material experiencing particularly widespread adoption [43].

### Detection

By bypassing culture, samples can be analyzed by qPCR in as few as three hours or stabilized for transport and extended storage prior to analysis [126], However, this analysis may detect residual signals from organisms that are not viable or infectious at the time of collection [132]. Although gene markers must be pre-specified, qPCR (alongside reverse-transcription PCR (RT-PCR) for RNA markers) provides a consistent approach for detecting targets ranging from FIB to viruses, human mitochondrial DNA, and genes conferring pathogenicity or antimicrobial resistance [27, 43, 133]. qPCR assays can also be multiplexed to detect a limited number of targets in a single reaction. Furthermore, the recent development of qPCR array cards that enable simultaneous detection of dozens of gene targets in a single sample demonstrates the growing feasibility of direct detection of a comprehensive set of enteric pathogens alongside functional genes and fecal indicators [134–142].

### Analytical Sensitivity

Although qPCR is a sensitive method relative to culture and conventional PCR [20, 143], it is vulnerable to interference from other substances common in environmental waters that can reduce the availability of target DNA or inhibit polymerase function, limiting assay sensitivity [144]. Strategies to mitigate matrix interference include sample dilution or chemical treatment, nucleic acid purification, inhibition-resistant reagents, and the use of multiple processing and internal controls to both identify inhibited samples and competitively bind interfering substances [144–146]. Such approaches increase the complexity, expense, and time requirements for analysis, and physical removal of inhibitors through dilution or purification also reduces target DNA, providing diminishing returns to analytical sensitivity [147, 148]. Complete abatement of qPCR inhibition is likely unrealistic; nevertheless, recent efforts to standardize qPCR procedures for water quality assessment suggest that a set of existing mitigation practices is sufficient to render matrix interference a manageable nuisance in most applications [81, 144, 149•]. Increasing adoption of digital PCR (dPCR), a quantitative PCR approach robust to inhibition, will likely further alleviate the challenge of inhibition for routine molecular detection of fecal microbes [61, 114, 128, 150–152].

Improved assay sensitivity offers little benefit if the target is unlikely to be present in the test sample due to low ambient concentrations. While simulation studies indicate that the concentrations at which bacterial indicators represent elevated risk of illness are well above the limits of detection [153], enteric viruses, protozoan parasites, and some alternative indicators (e.g., coliphages) commonly require larger sample volumes for reliable capture, necessitating concentration methods to obtain test sample volumes that can be accommodated by the chosen detection method [44, 128]. Filtration approaches that allow simultaneous concentration of a wide range of organisms are increasingly used to process samples, including as part of automated large-volume samplers, prior to culture or molecular detection [128, 154–156]. Ultrafiltration techniques in particular have demonstrated reasonably efficient and consistent recovery for a variety of organisms, water types, and sample volumes, providing a natural complement to multitarget arrays, and increasingly appear to be the default concentration approach for many applications [154, 157–161]. Co-concentration of qPCR inhibitors during ultrafiltration is a concern, but effective inhibition mitigation has been demonstrated by further processing of the concentrate prior to analysis [160, 162–164].

### Data Quality

Generalized data reporting guidelines notwithstanding [165], differences in analytical procedures and data handling practices were identified as major sources of variability in a multilaboratory comparison study of primarily qPCR-based MST approaches [24, 166]. Substantial effort has been devoted in recent years to the development and implementation of standardized protocols and quality control metrics for fecal indicator assessment by qPCR [113, 167, 168]. A notable feature shared by these protocols and other recent recommendations for improved reliability of molecular detection methods is a reliance on numerous controls throughout the procedure [128, 137]. While the use of positive and negative controls is standard for most analytical techniques, requirements outlined in standardized qPCR protocols generally include multiple serial dilutions of standard reference material to construct calibration curves, sample processing controls (SPC), method extraction blanks (MEB), internal amplification controls (IAC), and no template controls (NTC), with two or three replicates of each sample, control, and standard dilution series concentration analyzed on each instrument run [113]. Each additional reference or control material must be obtained, prepared, stored, and used in the appropriate manner, increasing per-sample costs and introducing considerable complexity and opportunity for user error. In a recent large demonstration project, some laboratories without extensive previous qPCR experience struggled to achieve adequate quality control despite receiving method-specific instrumentation, materials, and training. Even laboratories with substantial qPCR experience regularly failed to meet data quality criteria in this study [149•]. The compounding complexity required for reliable results suggests that qPCR in its current form may be unsuitable for routine monitoring purposes except in particularly well-resourced laboratories that regularly process sufficient sample numbers to warrant the equipment, properly maintain assay materials, and ensure sustained institutional experience. By contrast, culture-based FIB detection has grown increasingly accessible following recent efforts to develop low-cost field tests that can be performed with minimal equipment at ambient temperatures [125, 169, 170]. While still requiring substantial resources and expertise, dPCR requires fewer controls and precise reference materials than qPCR because it is robust to matrix interference and offers absolute quantification, features which may position dPCR to be increasingly adopted for general use [171]. Both up-front and per-reaction costs are considerably higher for dPCR compared to qPCR, but the improved multiplexing performance, fewer required control reactions, and greater precision of dPCR present opportunities to mitigate differences in per-sample costs [114, 143, 172, 173].

## Health Relevance and Protection

In addition to revealing fecal pollution and elucidating its sources, fecal indicators are widely used to characterize health hazards in waters potentially impacted by fecal contamination. This approach has proven somewhat effective in drinking water and for recreational exposures during wet weather or near point sources of fecal pollution [25, 28, 35]. A recent review found increased likelihood of co-detection of fecal indicators and enteric pathogens in recreational waters under similar conditions [27]. However, relationships between fecal indicators and gastrointestinal illness have mostly not been observed in waters impacted by non-point source pollution [25, 33, 174], despite well-documented risks to swimmers [31].

In the absence of consistent empirical relationships, quantitative microbial risk assessment (QMRA) has been used to estimate the health implications of various indicators introduced by different fecal sources [153, 175]. Notably, threshold concentrations at which MST markers correspond to increased risk of illness have been estimated in several QMRA studies; these thresholds are comfortably above the typical limits of detection, suggesting that the markers are highly likely to be detected should their associated pathogens be present at hazardous levels [2•, 3, 49, 56, 62, 176].

Indicator-based risk assessment requires defining the relationships between the indicator concentration and the index pathogens selected for consideration, typically a subset of pathogens expected to account for the majority of the risk and for which dose-response relationships have been characterized [55, 175]. A substantial body of research characterizing processes affecting indicator-pathogen relationships has culminated in the recent publication of several comprehensive reviews and meta-analyses of the occurrence, transport, and persistence of indicators and common index pathogens in fecal waste streams and surface water [17, 27, 177–179]. Associations between indicators and pathogens in surface water have been largely inconsistent, although empirical determination of these relationships is challenged by the limitations of direct pathogen detection; associations are more commonly observed among more frequently detected organisms [27, 36]. Microbial occurrence is more consistent in feces and particularly in sewage, which smooths the high individual variability in fecal microbe shedding by representing the combined fecal inputs of populations [177, 178, 180–182]. Despite less frequent detection of alternative indicators in recreational waters [27], high concentrations of multiple human-associated markers have been reported worldwide in both raw and biologically treated wastewater [83•]. Meta-analyses have also found high coliphage and norovirus densities in raw sewage around the world [178, 180]. A wide range of pathogens have frequently been detected in stormwater, though with greater variability and typically at lower concentrations than in sewage [179].

Upon introduction to the environment, microbial contaminants are subject to highly variable dispersal and decay processes [17]. Differential transport and decay of indicators and pathogens reduce associations between them that may have been present at the source, but the numerous factors affecting environmental fate and transport were previously poorly understood [24]. Many studies have since investigated the persistence of different organisms under various conditions, often using seeded mesocosms [17]. A recent QMRA study incorporated a meta-analysis of decay rates and found that the risk represented by a particular concentration of sewage-derived HF183 increased with time because it decayed faster than norovirus, the principal driver of risk [2•]. Conversely, another QMRA found that failing to account for differential decay overestimated the risk posed by animal fecal sources but did not meaningfully affect the risk from human sources, which in this study was dominated by viruses with similar decay characteristics as human-associated markers [49]. However, the multitude of factors that affect microbial fate and transport calls into question the generalizability of such assessments given the wide spatial and temporal variation in natural conditions, particularly across organisms and fecal sources [17, 183].

## Applications and Recommendations

The use of FIB for fecal contamination assessment continues to have many applications and has expanded with the broad adoption of MST approaches. Routine monitoring of surface waters is widely conducted in order to assess regulatory compliance, characterize water quality trends, and provide timely warnings to protect public health [184–189]. Specific investigations, often supplemented with historical monitoring data, may be conducted to inform management strategies and remediation efforts and to evaluate the impacts of infrastructure, policy, and practices [59, 118•, 179, 190–193]. Forensic applications are increasingly pursued to assign responsibility for fecal pollution, largely enabled by wider adoption of MST approaches and molecular detection methods [13, 69, 70, 106, 117•, 120, 123].

Despite their widespread use, evidence for the suitability of indicators in evaluative applications remains mixed and appears to vary depending on the timing and conditions under which they are applied. Under favorable conditions that provide more proximate connections between indicators and their sources (e.g., near wastewater outfalls or in household drinking water), indicator abundance may be associated with increased risk of illness that one would expect with elevated fecal loads [25, 29, 194]. Interventions that directly impact sources, such as gull deterrence at beaches, may also be reflected in indicator concentrations [118•]. Contamination through less direct processes, such as non-point source pollution, is subject to the numerous factors affecting microbial fate and transport, which may account for the large temporal variability often observed in FIB concentrations and the lack of association with illness [25, 127, 195]. Such variability limits the amount of information conveyed by individual observations, requiring much larger datasets to disentangle trends in indicator occurrence from the inherent variance in indicator measurements [188, 189]. These limitations are especially pronounced when anticipated effects are indirect and small relative to typical indicator concentrations, which may be maintained in part by other sources and pathways of contamination [138, 192, 193, 196]. The outsized influence of precipitation on microbial concentrations may obscure less dramatic dynamics in many systems [195]. Furthermore, clear long-term indicator trends do not necessarily represent concomitant changes in pathogen hazards [157].

The increasing feasibility of comprehensive direct pathogen detection suggests that situations demanding a high degree of confidence about the presence of hazardous fecal contamination may be best served by assaying pathogens directly, utilizing concentration methods to improve sensitivity as appropriate [128]. The possibility of false negatives due to temporal and spatial variability, while partially addressable through strategies such as composite sampling, nevertheless suggests that general fecal indicators should continue to be assessed to complement direct pathogen detection efforts. Despite the recent introduction of procedures to simultaneously quantify multiple FIB, MST, and pathogen genes in under 4 hours [139], the expense, necessary expertise, and rapid pace of change likely preclude the routine application of direct pathogen detection for some time to come. Meanwhile, protecting public health in recreational waters remains an important (and legally mandated) goal. High-traffic beaches with established daily microbial water quality testing programs and dedicated laboratory facilities are likely to benefit from implementing rapid FIB qPCR monitoring with same-day notification [126, 197]. As such beaches are often located near large urban areas and impacted by human sources, they may further benefit from instead implementing simultaneous monitoring of FIB and human-associated markers by duplex dPCR to establish time trends in human-source contamination at little additional cost [112, 114]. Although associations between human-associated markers and gastrointestinal illness are generally lacking [32, 174], their regular application across multiple human-impacted locations may provide useful information to prioritize remediation efforts [111, 112].

Locations that host fewer recreators, have limited monitoring resources and sampling frequency, or are impacted by non-point sources, for which generalizable relationships between indicators and risk are lacking, are unlikely to realize similar benefits from adopting rapid molecular monitoring while incurring substantial additional expense, complexity, and opportunity for error [149•, 198, 199]. Rather, supplementing existing FIB monitoring programs with predictive modeling may present a more feasible approach for expanding the scope of microbial water quality assessment in the numerous surface waters for which monitoring resources are limited [200, 201]. Precipitation—perhaps the most consistent factor in recreational water quality, reliably increasing ambient fecal microbe concentrations and the risk of illness—likewise tends to drive predictive FIB model outcomes [119, 179, 195, 202–204]; for many applications, providing recreational guidance on the basis of recent precipitation may well present the most reliable method for protecting public health [110].

## Conclusions

The value of fecal indicators as investigative tools to identify fecal pollution has been reaffirmed and expanded with the broad adoption of MST approaches, despite imperfect sensitivity and specificity [58, 81, 110, 118•, 119]. Also, the literature on many technical aspects of fecal indicators and their applications has notably matured, as demonstrated by the recent publication of several comprehensive reviews [2•, 17, 25, 27, 44, 144, 153]. The improved understanding of microbial dynamics and detection approaches has supported the development of more nuanced and robust procedures for characterizing fecal pollution. Nevertheless, this body of work also serves to emphasize the incredible complexity and variability of fecal microbes in the environment and reinforces the challenges to their effective use.

Major challenges remain in source apportionment, risk characterization, and impact evaluation. Additional research is needed to further refine indicators of fecal contamination and to add tools to the toolbox appropriate for emerging challenges. New indicators are needed to detect antimicrobial-resistant bacteria and resistance genes in water samples and to link environmental antimicrobial resistance to health risks. Better risk characterizations are needed to improve risk modeling and to expand the timing and conditions under which these models can reliably predict threats to human health. Also, empirical models that identify associations between indicators and co-measured predictors, particularly rainfall, can likely alleviate some of the sample burden associated with water quality assessments. Direct pathogen detection is becoming more feasible than in previous years and is likely to be more of a focus for water quality tests in the future. Together, these advances are improving water quality assessments and identifying appropriate actions to safeguard public health across the globe.

## References

[1] ASCE (2017). Infrastructure report card - wastewater.

[2] Boehm AB, Graham KE, Jennings WC (2018). Can we swim yet? Systematic review, meta-analysis, and risk assessment of aging sewage in surface waters. Environ Sci Technol.

[3] McLellan SL, Sauer EP, Corsi SR, Bootsma MJ, Boehm AB, Spencer SK (2018). Sewage loading and microbial risk in urban waters of the Great Lakes. Elem Sci Anth.

[4] Kapoor V, Gupta I, Pasha ABMT, Phan D (2018). Real-time quantitative PCR measurements of fecal indicator bacteria and human-associated source tracking markers in a Texas river following Hurricane Harvey. Environ Sci Technol Lett.

[5] Yu P, Zaleski A, Li Q, He Y, Mapili K, Pruden A (2018). Elevated levels of pathogenic indicator bacteria and antibiotic resistance genes after Hurricane Harvey’s flooding in Houston. Environ Sci Technol Lett.

[6] Miller JD, Hutchins M (2017). The impacts of urbanisation and climate change on urban flooding and urban water quality: a review of the evidence concerning the United Kingdom. J Hydrol Reg Stud.

[7] CDC (2019). Antibiotic resistance threats in the United States, 2019.

[8] WHO (2015). Antimicrobial resistance: an emerging water, sanitation and hygiene issue. Brief.

[9] WHO. Antimicrobial resistance: global report on surveillance. Geneva, Switzerland: World Health Organization; 2014. https://www.who.int/antimicrobialresistance/publications/surveillancereport/en/.

[10] Lippi D, Gotuzzo E (2014). The greatest steps towards the discovery of Vibrio cholerae. Clin Microbiol Infect.

[11] Burian SJ, Nix SJ, Pitt RE, Rocky DS (2000). Urban wastewater management in the United States: past, present, and future. J Urban Technol.

[12] Freedman DA (1991). Statistical models and shoe leather. Sociol Methodol.

[13] Teaf CM, Flores D, Garber M, Harwood VJ (2018). Toward forensic uses of microbial source tracking. Microbiol Spectr.

[14] National Research Council (2004). Indicators for waterborne pathogens.

[15] WHO (2003). Guidelines for safe recreational water environments: coastal and fresh waters.

[16] Oliveira J, Cunha A, Castilho F, Romalde JL, Pereira MJ (2011). Microbial contamination and purification of bivalve shellfish: crucial aspects in monitoring and future perspectives - a mini-review. Food Control.

[17] Korajkic A, Wanjugi P, Brooks L, Cao Y, Harwood VJ. Persistence and decay of fecal microbiota in aquatic habitats. Microbiol Mol Biol Rev. 2019;83. 10.1128/MMBR.00005-19. 10.1128/MMBR.00005-19 PMC7405076 31578217

[18] Ashbolt NJ, Schoen ME, Soller JA, Roser DJ (2010). Predicting pathogen risks to aid beach management: the real value of quantitative microbial risk assessment (QMRA). Water Res.

[19] Gerba CP, Betancourt WQ, Kitajima M, Rock CM (2018). Reducing uncertainty in estimating virus reduction by advanced water treatment processes. Water Res.

[20] Liu J, Kabir F, Manneh J, Lertsethtakarm P, Begum S, Gratz J (2014). Development and assessment of molecular diagnostic tests for 15 enteropathogens causing childhood diarrhoea: a multicentre study. Lancet Infect Dis.

[21] Field KG, Samadpour M (2007). Fecal source tracking, the indicator paradigm, and managing water quality. Water Res.

[22] Stewart JR, Gast RJ, Fujioka RS, Solo-Gabriele HM, Meschke JS, Amaral-Zettler LA (2008). The coastal environment and human health: microbial indicators, pathogens, sentinels and reservoirs. Environ Health.

[23] Stoeckel DM, Harwood VJ (2007). Performance, design, and analysis in microbial source tracking studies. Appl Environ Microbiol.

[24] Stewart JR, Boehm AB, Dubinsky EA, Fong T-T, Goodwin KD, Griffith JF (2013). Recommendations following a multi-laboratory comparison of microbial source tracking methods. Water Res.

[25] Fewtrell L, Kay D (2015). Recreational water and infection: a review of recent findings. Curr Environ Health Rep.

[26] Sclar GD, Penakalapati G, Amato HK, Garn JV, Alexander K, Freeman MC (2016). Assessing the impact of sanitation on indicators of fecal exposure along principal transmission pathways: a systematic review. Int J Hyg Environ Health.

[27] Korajkic A, McMinn B, Harwood V (2018). Relationships between microbial indicators and pathogens in recreational water settings. Int J Environ Res Public Health.

[28] Gruber JS, Ercumen A, Colford JM (2014). Coliform bacteria as indicators of diarrheal risk in household drinking water: systematic review and meta-analysis. PLoS One.

[29] Ercumen A, Arnold BF, Naser AM, Unicomb L, Colford JM, Luby SP (2017). Potential sources of bias in the use of Escherichia coli to measure waterborne diarrhoea risk in low-income settings. Trop Med Int Health.

[30] Luby SP, Halder AK, Huda TM, Unicomb L, Islam MS, Arnold BF (2015). Microbiological contamination of drinking water associated with subsequent child diarrhea. Am J Trop Med Hyg.

[31] Leonard AFC, Singer A, Ukoumunne OC, Gaze WH, Garside R (2018). Is it safe to go back into the water? A systematic review and meta-analysis of the risk of acquiring infections from recreational exposure to seawater. Int J Epidemiol.

[32] Griffith JF, Weisberg SB, Arnold BF, Cao Y, Schiff KC, Colford JM (2016). Epidemiologic evaluation of multiple alternate microbial water quality monitoring indicators at three California beaches. Water Res.

[33] Benjamin-Chung J, Arnold BF, Wade TJ, Schiff K, Griffith JF, Dufour AP (2017). Coliphages and gastrointestinal illness in recreational waters. Epidemiology..

[34] Colford JM, Schiff KC, Griffith JF, Yau V, Arnold BF, Wright CC (2012). Using rapid indicators for Enterococcus to assess the risk of illness after exposure to urban runoff contaminated marine water. Water Res.

[35] Wade TJ, Pai N, Eisenberg JNS, Colford JM. Do U.S. Environmental protection agency water quality guidelines for recreational waters prevent gastrointestinal illness? A systematic review and meta-analysis. Environ Health Perspect 2003;111:1102–1109. doi:10.1289/ehp.6241 10.1289/ehp.6241 PMC1241558 12826481

[36] Wu J, Long SC, Das D, Dorner SM (2011). Are microbial indicators and pathogens correlated? A statistical analysis of 40 years of research. J Water Health.

[37] Byappanahalli M, Fowler M, Shively D, Whitman R (2003). Ubiquity and persistence of Escherichia coli in a Midwestern coastal stream. Appl Environ Microbiol.

[38] Byappanahalli M, Fujioka R (2004). Indigenous soil bacteria and low moisture may limit but allow faecal bacteria to multiply and become a minor population in tropical soils. Water Sci Technol.

[39] Oh S, Buddenborg S, Yoder-Himes DR, Tiedje JM, Konstantinidis KT (2012). Genomic diversity of Escherichia isolates from diverse habitats. PLoS One.

[40] Rivera SC, Tc H, Ga T (1988). Isolation of fecal coliform from pristine sites in a tropical rain forest. Appl Environ Microbiol.

[41] Solo-Gabriele HM, Wolfert MA, Desmarais TR, Palmer CJ (2000). Sources of Escherichia coli in a coastal subtropical environment. Appl Environ Microbiol.

[42] Ishii S, Sadowsky MJ (2008). Escherichia coli in the environment: implications for water quality and human health. Microbes Environ.

[43] Harwood VJ, Staley C, Badgley BD, Borges K, Korajkic A (2014). Microbial source tracking markers for detection of fecal contamination in environmental waters: relationships between pathogens and human health outcomes. FEMS Microbiol Rev.

[44] McMinn BR, Ashbolt NJ, Korajkic A (2017). Bacteriophages as indicators of faecal pollution and enteric virus removal. Lett Appl Microbiol.

[45] Vergara GGRV, Goh SG, Rezaeinejad S, Chang SY, Sobsey MD, Gin KYH (2015). Evaluation of FRNA coliphages as indicators of human enteric viruses in a tropical urban freshwater catchment. Water Res.

[46] Boehm AB, Van De Werfhorst LC, Griffith JF, Holden PA, Jay JA, Shanks OC (2013). Performance of forty-one microbial source tracking methods: a twenty-seven lab evaluation study. Water Res.

[47] Schoen ME, Soller JA, Ashbolt NJ (2011). Evaluating the importance of faecal sources in human-impacted waters. Water Res.

[48] Soller JA, Schoen ME, Bartrand T, Ravenscroft JE, Ashbolt NJ (2010). Estimated human health risks from exposure to recreational waters impacted by human and non-human sources of faecal contamination. Water Res.

[49] Wu B, Chunwei W, Zhang C, Sadowsky MJ, Dzakpasu M, Wang XC. Source-associated gastroenteritis risk from swimming exposure to aging fecal pathogens. Environ Sci Technol. 2019. 10.1021/acs.est.9b01188. 10.1021/acs.est.9b01188 31800232

[50] Soller JA, Schoen ME, Varghese A, Ichida AM, Boehm AB, Eftim S (2014). Human health risk implications of multiple sources of faecal indicator bacteria in a recreational waterbody. Water Res.

[51] Soller J, Bartrand T, Ravenscroft J, Molina M, Whelan G, Schoen M (2015). Estimated human health risks from recreational exposures to stormwater runoff containing animal faecal material. Environ Model Softw.

[52] Troeger C, Blacker BF, Khalil IA, Rao PC, Cao S, Zimsen SR (2018). Estimates of the global, regional, and national morbidity, mortality, and aetiologies of diarrhoea in 195 countries: a systematic analysis for the Global Burden of Disease Study 2016. Lancet Infect Dis.

[53] Liu J, Platts-Mills JA, Juma J, Kabir F, Nkeze J, Okoi C (2016). Use of quantitative molecular diagnostic methods to identify causes of diarrhoea in children: a reanalysis of the GEMS case-control study. Lancet..

[54] Platts-Mills JA, Babji S, Bodhidatta L, Gratz J, Haque R, Havt A (2015). Pathogen-specific burdens of community diarrhoea in developing countries: a multisite birth cohort study (MAL-ED). Lancet Glob Health.

[55] Sunger N, Hamilton KA, Morgan PM, Haas CN. Comparison of pathogen-derived ‘total risk’ with indicator-based correlations for recreational (swimming) exposure. Environ Sci Pollut Res. 2018. 10.1007/s11356-018-1881-x. 10.1007/s11356-018-1881-x 29644614

[56] Boehm AB, Soller JA, Shanks OC (2015). Human-associated fecal quantitative polymerase chain reaction measurements and simulated risk of gastrointestinal illness in recreational waters contaminated with raw sewage. Environ Sci Technol Lett.

[57] Delahoy MJ, Wodnik B, McAliley L, Penakalapati G, Swarthout J, Freeman MC, et al. Pathogens transmitted in animal feces in low- and middle-income countries. Int J Hyg Environ Health. 2018. 10.1016/j.ijheh.2018.03.005. 10.1016/j.ijheh.2018.03.005 PMC6013280 29729998

[58] Fuhrmeister ER, Ercumen A, Pickering AJ, Jeanis KM, Ahmed M, Brown S (2019). Predictors of enteric pathogens in the domestic environment from human and animal sources in rural Bangladesh. Environ Sci Technol.

[59] Odagiri M, Schriewer A, Daniels ME, Wuertz S, Smith WA, Clasen T (2016). Human fecal and pathogen exposure pathways in rural Indian villages and the effect of increased latrine coverage. Water Res.

[60] Penakalapati G, Swarthout J, Delahoy MJ, McAliley L, Wodnik B, Levy K (2017). Exposure to animal feces and human health: a systematic review and proposed research priorities. Environ Sci Technol.

[61] Steele JA, Blackwood AD, Griffith JF, Noble RT, Schiff KC (2018). Quantification of pathogens and markers of fecal contamination during storm events along popular surfing beaches in San Diego, California. Water Res.

[62] Brown KI, Graham KE, Boehm AB (2017). Risk-based threshold of gull-associated fecal marker concentrations for recreational water. Environ Sci Technol Lett.

[63] Budge S, Hutchings P, Parker A, Tyrrel S, Tulu T, Gizaw M (2019). Do domestic animals contribute to bacterial contamination of infant transmission pathways? Formative evidence from Ethiopia. J Water Health.

[64] Reid B, Orgle J, Roy K, Pongolani C, Chileshe M, Stoltzfus R. Characterizing potential risks of fecal–oral microbial transmission for infants and young children in rural Zambia. Am J Trop Med Hyg. 2018. 10.4269/ajtmh.17-0124. 10.4269/ajtmh.17-0124 PMC5930879 29405109

[65] Bernhard AE, Field KG. A PCR Assay To discriminate human and ruminant feces on the basis of host differences in bacteroides-prevotella genes encoding 16S rRNA. Appl Environ Microbiol. 2000;66:4571–4. 10.1128/AEM.66.10.4571-4574.2000. 10.1128/aem.66.10.4571-4574.2000 PMC92346 11010920

[66] Ahmed W, Hughes B, Harwood V (2016). Current status of marker genes of bacteroides and related taxa for identifying sewage pollution in environmental waters. Water..

[67] Ahmed W, Gyawali P, Feng S, McLellan SL. Host specificity and sensitivity of established and novel sewage-associated marker genes in human and nonhuman fecal samples. Appl Environ Microbiol. 2019;85. 10.1128/AEM.00641-19. 10.1128/AEM.00641-19 PMC6606881 31076423

[68] Ahmed W, Payyappat S, Cassidy M, Besley C, Power K (2018). Novel crAssphage marker genes ascertain sewage pollution in a recreational lake receiving urban stormwater runoff. Water Res.

[69] Harris AR, Pickering AJ, Harris M, Doza S, Islam MS, Unicomb L, et al. Ruminants contribute fecal contamination to the urban household environment in Dhaka, Bangladesh. Environ Sci Technol. 2016;50:4642–4649. doi:10.1021/acs.est.5b06282 10.1021/acs.est.5b06282 27045990

[70] Boehm AB, Wang D, Ercumen A, Shea M, Harris AR, Shanks OC, et al. Occurrence of host-associated fecal markers on child hands, household soil, and drinking water in rural Bangladeshi households. Environ Sci Technol Lett. 2016. 10.1021/acs.estlett.6b00382. PMC7326215 32607385

[71] Symonds EM, Young S, Verbyla ME, McQuaig-Ulrich SM, Ross E, Jiménez JA (2017). Microbial source tracking in shellfish harvesting waters in the Gulf of Nicoya, Costa Rica. Water Res.

[72] Odagiri M, Schriewer A, Hanley K, Wuertz S, Misra PR, Panigrahi P (2015). Validation of Bacteroidales quantitative PCR assays targeting human and animal fecal contamination in the public and domestic domains in India. Sci Total Environ.

[73] Haramoto E, Osada R (2018). Assessment and application of host-specific Bacteroidales genetic markers for microbial source tracking of river water in Japan. PLoS One.

[74] Malla B, Makise K, Nakaya K, Mochizuki T, Yamada T, Haramoto E (2019). Evaluation of Human- and animal-specific viral markers and application of CrAssphage, pepper mild mottle virus, and tobacco mosaic virus as potential fecal pollution markers to river water in Japan. Food Environ Virol.

[75] Holcomb DA, Knee J, Sumner T, Adriano Z, de Bruijn E, Nalá R (2020). Human fecal contamination of water, soil, and surfaces in households sharing poor-quality sanitation facilities in Maputo, Mozambique. Int J Hyg Environ Health.

[76] Gyawali P, Croucher D, Ahmed W, Devane M, Hewitt J (2019). Evaluation of pepper mild mottle virus as an indicator of human faecal pollution in shellfish and growing waters. Water Res.

[77] Malla B, Ghaju Shrestha R, Tandukar S, Bhandari D, Inoue D, Sei K (2018). Validation of host-specific Bacteroidales quantitative PCR assays and their application to microbial source tracking of drinking water sources in the Kathmandu Valley, Nepal. J Appl Microbiol.

[78] Malla B, Ghaju Shrestha R, Tandukar S, Sherchand JB, Haramoto E (2019). Performance evaluation of human-specific viral markers and application of pepper mild mottle virus and CrAssphage to environmental water samples as fecal pollution markers in the Kathmandu Valley, Nepal. Food Environ Virol.

[79] Nshimyimana JP, Cruz MC, Thompson RJ, Wuertz S (2017). Bacteroidales markers for microbial source tracking in Southeast Asia. Water Res.

[80] Somnark P, Chyerochana N, Mongkolsuk S, Sirikanchana K (2018). Performance evaluation of Bacteroidales genetic markers for human and animal microbial source tracking in tropical agricultural watersheds. Environ Pollut.

[81] Li X, Sivaganesan M, Kelty CA, Zimmer-Faust A, Clinton P, Reichman JR (2019). Large-scale implementation of standardized quantitative real-time PCR fecal source identification procedures in the Tillamook Bay Watershed. PLoS One.

[82] Ahmed W, Lobos A, Senkbeil J, Peraud J, Gallard J, Harwood VJ (2018). Evaluation of the novel crAssphage marker for sewage pollution tracking in storm drain outfalls in Tampa, Florida. Water Res.

[83] Mayer RE, Reischer GH, Ixenmaier SK, Derx J, Blaschke AP, Ebdon JE (2018). Global distribution of human-associated fecal genetic markers in reference samples from six continents. Environ Sci Technol.

[84] Feng S, Bootsma M, McLellan SL. Human-associated Lachnospiraceae genetic markers improve detection of fecal pollution sources in urban waters. Appl Environ Microbiol. 2018;84. 10.1128/AEM.00309-18. 10.1128/AEM.00309-18 PMC6029095 29728386

[85] Stachler E, Kelty C, Sivaganesan M, Li X, Bibby K, Shanks OC (2017). Quantitative CrAssphage PCR assays for human fecal pollution measurement. Environ Sci Technol.

[86] Cinek O, Mazankova K, Kramna L, Odeh R, Alassaf A, Ibekwe MU (2018). Quantitative CrAssphage real-time PCR assay derived from data of multiple geographically distant populations. J Med Virol.

[87] Liang Y, Jin X, Huang Y, Chen S (2018). Development and application of a real-time polymerase chain reaction assay for detection of a novel gut bacteriophage (crAssphage). J Med Virol.

[88] García-Aljaro C, Ballesté E, Muniesa M, Jofre J (2017). Determination of crAssphage in water samples and applicability for tracking human faecal pollution. Microb Biotechnol.

[89] Ahmed W, Payyappat S, Cassidy M, Besley C (2019). A duplex PCR assay for the simultaneous quantification of Bacteroides HF183 and crAssphage CPQ_056 marker genes in untreated sewage and stormwater. Environ Int.

[90] Gomi R, Matsuda T, Matsui Y, Yoneda M (2014). Fecal source tracking in water by next-generation sequencing technologies using host-specific Escherichia coli genetic markers. Environ Sci Technol.

[91] Warish A, Triplett C, Gomi R, Gyawali P, Hodgers L, Toze S (2015). Assessment of genetic markers for tracking the sources of human wastewater associated Escherichia coli in environmental waters. Environ Sci Technol.

[92] Senkbeil JK, Ahmed W, Conrad J, Harwood VJ (2019). Use of Escherichia coli genes associated with human sewage to track fecal contamination source in subtropical waters. Sci Total Environ.

[93] Hughes B, Beale DJ, Dennis PG, Cook S, Ahmed W (2017). Cross-comparison of human wastewater-associated molecular markers in relation to fecal indicator bacteria and enteric viruses in recreational beach waters. Appl Environ Microbiol.

[94] Feng S, McLellan SL (2019). Highly specific sewage-derived Bacteroides quantitative PCR assays target sewage-polluted waters. Appl Environ Microbiol.

[95] McLellan SL, Eren AM (2014). Discovering new indicators of fecal pollution. Trends Microbiol.

[96] Dutilh BE, Cassman N, McNair K, Sanchez SE, Silva GGZ, Boling L (2014). A highly abundant bacteriophage discovered in the unknown sequences of human faecal metagenomes. Nat Commun.

[97] Unno T, Staley C, Brown CM, Han D, Sadowksy MJ, Hur H-G. Fecal pollution: new trends and challenges in microbial source tracking using next-generation-sequencing. Environ Microbiol 2018;0. doi:10.1111/1462-2920.14281 10.1111/1462-2920.14281 29797757

[98] O’Dea C, Zhang Q, Staley C, Masters N, Kuballa A, Fisher P (2019). Compositional and temporal stability of fecal taxon libraries for use with SourceTracker in sub-tropical catchments. Water Res.

[99] Staley C, Kaiser T, Lobos A, Ahmed W, Harwood VJ, Brown CM (2018). Application of SourceTracker for accurate identification of fecal pollution in recreational freshwater: a double-blinded study. Environ Sci Technol.

[100] Hägglund M, Bäckman S, Macellaro A, Lindgren P, Borgmästars E, Jacobsson K (2018). Accounting for bacterial overlap between raw water communities and contaminating sources improves the accuracy of signature-based microbial source tracking. Front Microbiol.

[101] Brown CM, Mathai PP, Loesekann T, Staley C, Sadowsky MJ (2019). Influence of library composition on SourceTracker predictions for community-based microbial source tracking. Environ Sci Technol.

[102] Bauza V, Madadi VO, Ocharo RM, Nguyen TH, Guest JS (2019). Microbial source tracking using 16S rRNA amplicon sequencing identifies evidence of widespread contamination from young children’s feces in an urban slum of Nairobi, Kenya. Environ Sci Technol.

[103] Hu YOO, Ndegwa N, Alneberg J, Johansson S, Logue JB, Huss M (2018). Stationary and portable sequencing-based approaches for tracing wastewater contamination in urban stormwater systems. Sci Rep.

[104] Acharya K, Khanal S, Pantha K, Amatya N, Davenport RJ, Werner D (2019). A comparative assessment of conventional and molecular methods, including MinION nanopore sequencing, for surveying water quality. Sci Rep.

[105] Ahmed W, Staley C, Sadowsky MJ, Gyawali P, Sidhu J, Palmer A (2015). Toolbox approaches using molecular markers and 16S rRNA gene amplicon data sets for identification of fecal pollution in surface water. Appl Environ Microbiol.

[106] Kirs M, Kisand V, Wong M, Caffaro-Filho RA, Moravcik P, Harwood VJ (2017). Multiple lines of evidence to identify sewage as the cause of water quality impairment in an urbanized tropical watershed. Water Res.

[107] Tillett BJ, Sharley D, Almeida MIGS, Valenzuela I, Hoffmann AA, Pettigrove V (2018). A short work-flow to effectively source faecal pollution in recreational waters – a case study. Sci Total Environ.

[108] Symonds EM, Nguyen KH, Harwood VJ, Breitbart M. Pepper mild mottle virus: a plant pathogen with a greater purpose in (waste)water treatment development and public health management. Water Res. 2018. 10.1016/j.watres.2018.06.066. 10.1016/j.watres.2018.06.066 PMC6162155 30005176

[109] Crank K, Petersen S, Bibby K (2019). Quantitative microbial risk assessment of swimming in sewage impacted waters using CrAssphage and pepper mild mottle virus in a customizable model. Environ Sci Technol Lett.

[110] Ahmed W, Payyappat S, Cassidy M, Besley C (2019). Enhanced insights from human and animal host-associated molecular marker genes in a freshwater lake receiving wet weather overflows. Sci Rep.

[111] Cao Y, Sivaganesan M, Kelty CA, Wang D, Boehm AB, Griffith JF (2018). A human fecal contamination score for ranking recreational sites using the HF183/BacR287 quantitative real-time PCR method. Water Res.

[112] Cao Y, Raith MR, Smith PD, Griffith JF, Weisberg SB, Schriewer A, et al. Regional assessment of human fecal contamination in Southern California coastal drainages. Int J Environ Res Public Health. 2017;14. 10.3390/ijerph14080874. 10.3390/ijerph14080874 PMC5580578 28777324

[113] Shanks OC, Kelty CA, Oshiro R, Haugland RA, Madi T, Brooks L (2016). Data acceptance criteria for standardized human-associated fecal source identification quantitative real-time PCR methods. Appl Environ Microbiol.

[114] Cao Y, Raith MR, Griffith JF (2015). Droplet digital PCR for simultaneous quantification of general and human-associated fecal indicators for water quality assessment. Water Res.

[115] Jenkins MW, Tiwari S, Lorente M, Gichaba CM, Wuertz S (2009). Identifying human and livestock sources of fecal contamination in Kenya with host-specific Bacteroidales assays. Water Res.

[116] Prendergast AJ, Gharpure R, Mor S, Viney M, Dube K, Lello J (2019). Putting the “A” into WaSH: a call for integrated management of water, animals, sanitation, and hygiene. Lancet Planet Health.

[117] Nguyen KHH, Senay C, Young S, Nayak B, Lobos A, Conrad J (2018). Determination of wild animal sources of fecal indicator bacteria by microbial source tracking (MST) influences regulatory decisions. Water Res.

[118] • Nevers MB, Byappanahalli MN, Shively D, Buszka PM, Jackson PR, Phanikumar MS. Identifying and eliminating sources of recreational water quality degradation along an urban coast. J Environ Qual. 2018. 10.2134/jeq2017.11.0461**This study used MST approaches not only to identify fecal sources but also to evaluate interventions implemented in response to the initial MST analysis, reporting substantial reductions in gull-associated markers under a gull deterrence program**. 10.2134/jeq2017.11.0461 30272790

[119] Dila DK, Corsi SR, Lenaker P, Baldwin AK, Bootsma MJ, McLellan S. Patterns of host-associated fecal indicators driven by hydrology, precipitation, and land use attributes in Great Lakes watersheds. Environ Sci Technol. 2018. 10.1021/acs.est.8b01945. 10.1021/acs.est.8b01945 PMC6437017 30192524

[120] Paruch L, Paruch AM, Sørheim R. DNA-based faecal source tracking of contaminated drinking water causing a large Campylobacter outbreak in Norway 2019. Int J Hyg Environ Health. 2019;113420. 10.1016/j.ijheh.2019.113420. 10.1016/j.ijheh.2019.113420 31748129

[121] Chen H, Bai X, Li Y, Jing L, Chen R, Teng Y. Source identification of antibiotic resistance genes in a peri-urban river using novel crAssphage marker genes and metagenomic signatures. Water Res. 2019;115098. 10.1016/j.watres.2019.115098. 10.1016/j.watres.2019.115098 31574349

[122] Larsson DGJ, Andremont A, Bengtsson-Palme J, Brandt KK, de Roda Husman AM, Fagerstedt P (2018). Critical knowledge gaps and research needs related to the environmental dimensions of antibiotic resistance. Environ Int.

[123] Heaney CD, Myers K, Wing S, Hall D, Baron D, Stewart JR (2015). Source tracking swine fecal waste in surface water proximal to swine concentrated animal feeding operations. Sci Total Environ.

[124] Weidhaas JL, Macbeth TW, Olsen RL, Harwood VJ (2011). Correlation of quantitative PCR for a poultry-specific brevibacterium marker gene with bacterial and chemical indicators of water pollution in a watershed impacted by land application of poultry litter. Appl Environ Microbiol.

[125] Bain R, Bartram J, Elliott M, Matthews R, Mcmahan L, Tung R (2012). A summary catalogue of microbial drinking water tests for low and medium resource settings. Int J Environ Res Public Health.

[126] Dorevitch S, Shrestha A, DeFlorio-Barker S, Breitenbach C, Heimler I (2017). Monitoring urban beaches with qPCR vs. culture measures of fecal indicator bacteria: implications for public notification. Environ Health.

[127] Wyer MD, Kay D, Morgan H, Naylor S, Clark S, Watkins J, et al. Within-day variability in microbial concentrations at a UK designated bathing water: Implications for regulatory monitoring and the application of predictive modelling based on historical compliance data. Water Res X. 2018. 10.1016/j.wroa.2018.10.003. 10.1016/j.wroa.2018.10.003 PMC6549935 31193990

[128] Haramoto E, Kitajima M, Hata A, Torrey JR, Masago Y, Sano D (2018). A review on recent progress in the detection methods and prevalence of human enteric viruses in water. Water Res.

[129] Bonadonna L, Briancesco R, La Rosa G (2019). Innovative analytical methods for monitoring microbiological and virological water quality. Microchem J.

[130] Bigham T, Dooley JSG, Ternan NG, Snelling WJ, Héctor Castelán MC, Davis J. Assessing microbial water quality: electroanalytical approaches to the detection of coliforms. TrAC Trends Anal Chem. 2019;115670. 10.1016/j.trac.2019.115670.

[131] Gorski L, Rivadeneira P, Cooley MB. New strategies for the enumeration of enteric pathogens in water. Environ Microbiol Rep. 2019:1758–2229.12786. 10.1111/1758-2229.12786. 10.1111/1758-2229.12786 31342654

[132] Emerson JB, Adams RI, Román CMB, Brooks B, Coil DA, Dahlhausen K (2017). Schrödinger’s microbes: tools for distinguishing the living from the dead in microbial ecosystems. Microbiome.

[133] Stachler E, Crank K, Bibby K. Co-occurrence of crAssphage with antibiotic resistance genes in an impacted urban watershed. Environ Sci Technol Lett. 2019. 10.1021/acs.estlett.9b00130.

[134] Ahmed W, Zhang Q, Ishii S, Hamilton K, Haas C (2018). Microfluidic quantification of multiple enteric and opportunistic bacterial pathogens in roof-harvested rainwater tank samples. Environ Monit Assess.

[135] Ishii S, Nakamura T, Ozawa S, Kobayashi A, Sano D, Okabe S (2014). Water quality monitoring and risk assessment by simultaneous multipathogen quantification. Environ Sci Technol.

[136] Byappanahalli M, Nevers MB, Whitman RL, Ishii S. Application of microfluidic QPCR (MFQPCR) technique to monitor bacterial pathogens in beach water and complex environmental matrices. Environ Sci Technol Lett. 2015. 10.1021/acs.estlett.5b00251.

[137] Zhang Q, Ishii S (2018). Improved simultaneous quantification of multiple waterborne pathogens and fecal indicator bacteria with the use of a sample process control. Water Res.

[138] Baker KK, Senesac R, Sewell D, Sen Gupta A, Cumming O, Mumma J (2018). Fecal fingerprints of enteric pathogen contamination in public environments of Kisumu, Kenya, associated with human sanitation conditions and domestic animals. Environ Sci Technol.

[139] Shahraki AH, Heath D, Chaganti SR. Recreational water monitoring: nanofluidic qRT-PCR chip for assessing beach water safety. Environ DNA. 2019. 10.1002/edn3.30.

[140] Sadik NJ, Uprety SR, Nalweyiso A, Kiggundu N, Banadda NE, Shisler JL, et al. Quantification of multiple waterborne pathogens in drinking water, drainage channels, and surface water in Kampala, Uganda during seasonal variation. GeoHealth. 2017:258–69. 10.1002/2017GH000081. 10.1002/2017GH000081 PMC7007170 32158991

[141] Liu J, Gratz J, Amour C, Nshama R, Walongo T, Maro A (2016). Optimization of quantitative PCR methods for enteropathogen detection. PLoS One.

[142] Pholwat S, Liu J, Taniuchi M, Chinli R, Pongpan T, Thaipisutikul I (2019). Genotypic antimicrobial resistance assays for use on E. coli isolates and stool specimens. PLoS One.

[143] Riedel TE, Zimmer-Faust AG, Thulsiraj V, Madi T, Hanley KT, Ebentier DL (2014). Detection limits and cost comparisons of human- and gull-associated conventional and quantitative PCR assays in artificial and environmental waters. J Environ Manag.

[144] Nappier SP, Ichida A, Jaglo K, Haugland R, Jones KR (2019). Advancements in mitigating interference in quantitative polymerase chain reaction (qPCR) for microbial water quality monitoring. Sci Total Environ.

[145] Cao Y, Griffith JF, Dorevitch S, Weisberg SB (2012). Effectiveness of qPCR permutations, internal controls and dilution as means for minimizing the impact of inhibition while measuring Enterococcus in environmental waters. J Appl Microbiol.

[146] Haugland RA, Siefring S, Lavender J, Varma M (2012). Influences of sample interference and interference controls on quantification of enterococci fecal indicator bacteria in surface water samples by the qPCR method. Water Res.

[147] Cox AM, Goodwin KD (2013). Sample preparation methods for quantitative detection of DNA by molecular assays and marine biosensors. Mar Pollut Bull.

[148] McKee AM, Spear SF, Pierson TW (2015). The effect of dilution and the use of a post-extraction nucleic acid purification column on the accuracy, precision, and inhibition of environmental DNA samples. Biol Conserv.

[149] Aw TG, Sivaganesan M, Briggs S, Dreelin E, Aslan A, Dorevitch S (2019). Evaluation of multiple laboratory performance and variability in analysis of recreational freshwaters by a rapid Escherichia coli qPCR method (Draft Method C). Water Res.

[150] Nshimyimana JP, Cruz MC, Wuertz S, Thompson JR. Variably improved microbial source tracking with digital droplet PCR. Water Res. 2019. 10.1016/j.watres.2019.04.056. 10.1016/j.watres.2019.04.056 31096066

[151] Wang D, Yamahara KM, Cao Y, Boehm AB (2016). Absolute quantification of enterococcal 23S rRNA gene using digital PCR. Environ Sci Technol.

[152] Monteiro S, Santos R (2017). Nanofluidic digital PCR for the quantification of Norovirus for water quality assessment. PLoS One.

[153] Zhang Q, Gallard J, Wu B, Harwood VJ, Sadowsky MJ, Hamilton KA (2019). Synergy between quantitative microbial source tracking (qMST) and quantitative microbial risk assessment (QMRA): a review and prospectus. Environ Int.

[154] McMinn BR, Huff EM, Rhodes ER, Korajkic A (2017). Concentration and quantification of somatic and F+ coliphages from recreational waters. J Virol Methods.

[155] Lenaker PL, Corsi SR, Borchardt MA, Spencer SK, Baldwin AK, Lutz MA (2017). Hydrologic, land cover, and seasonal patterns of waterborne pathogens in Great Lakes tributaries. Water Res.

[156] Francy DS, Stelzer EA, Brady AMG, Huitger C, Bushon RN, Ip HS (2013). Comparison of filters for concentrating microbial indicators and pathogens in lake water samples. Appl Environ Microbiol.

[157] Weidhaas J, Anderson A, Jamal R. Elucidating waterborne pathogen presence and aiding source apportionment in an impaired stream. Appl Environ Microbiol. 2018;303. 10.1128/AEM.02510-17. 10.1128/AEM.02510-17 PMC5835731 29305503

[158] Li X, Harwood VJ, Nayak B, Weidhaas JL (2016). Ultrafiltration and microarray for detection of microbial source tracking marker and pathogen genes in riverine and marine systems. Appl Environ Microbiol.

[159] McGinnis S, Spencer S, Firnstahl A, Stokdyk J, Borchardt M, McCarthy DT (2018). Human Bacteroides and total coliforms as indicators of recent combined sewer overflows and rain events in urban creeks. Sci Total Environ.

[160] Ahmed W, Gyawali P, Toze S (2016). Evaluation of glass wool filters and hollow-fiber ultrafiltration concentration methods for qPCR detection of human adenoviruses and polyomaviruses in river water. Water Air Soil Pollut.

[161] Kahler A, Johnson T, Hahn D, Narayanan J, Derado G, Hill V (2015). Evaluation of an ultrafiltration-based procedure for simultaneous recovery of diverse microbes in source waters. Water..

[162] Mull B, Hill VR (2012). Recovery of diverse microbes in high turbidity surface water samples using dead-end ultrafiltration. J Microbiol Methods.

[163] Hill VR, Mull B, Jothikumar N, Ferdinand K, Vinjé J (2010). Detection of GI and GII noroviruses in ground water using ultrafiltration and TaqMan real-time RT-PCR. Food Environ Virol.

[164] Borgmästars E, Jazi MM, Persson S, Jansson L, Rådström P, Simonsson M (2017). Improved detection of norovirus and hepatitis A virus in surface water by applying pre-PCR processing. Food Environ Virol.

[165] Bustin SA, Benes V, Garson JA, Hellemans J, Huggett J, Kubista M (2009). The MIQE guidelines: minimum information for publication of quantitative real-time PCR experiments. Clin Chem.

[166] Ebentier DL, Hanley KT, Cao Y, Badgley BD, Boehm AB, Ervin JS (2013). Evaluation of the repeatability and reproducibility of a suite of qPCR-based microbial source tracking methods. Water Res.

[167] Haugland RA, Siefring SD, Varma M, Dufour AP, Brenner KP, Wade TJ (2014). Standardization of enterococci density estimates by EPA qPCR methods and comparison of beach action value exceedances in river waters with culture methods. J Microbiol Methods.

[168] Sivaganesan M, Aw TG, Briggs S, Dreelin E, Aslan A, Dorevitch S (2019). Standardized data quality acceptance criteria for a rapid Escherichia coli qPCR method (draft method C) for water quality monitoring at recreational beaches. Water Res.

[169] Brown J, Bir A, Bain RES (2020). Novel methods for global water safety monitoring: comparative analysis of low-cost, field-ready E. coli assays. npj Clean Water.

[170] Wang A, McMahan L, Rutstein S, Stauber C, Reyes J, Sobsey MD (2017). Household microbial water quality testing in a Peruvian demographic and health survey: evaluation of the compartment bag test for Escherichia coli. Am J Trop Med Hyg.

[171] Huggett JF, Foy CA, Benes V, Emslie K, Garson JA, Haynes R (2013). The digital MIQE guidelines: minimum information for publication of quantitative digital PCR experiments. Clin Chem.

[172] Yang R, Paparini A, Monis P, Ryan U (2014). Comparison of next-generation droplet digital PCR (ddPCR) with quantitative PCR (qPCR) for enumeration of Cryptosporidium oocysts in faecal samples. Int J Parasitol.

[173] Baker M (2012). Digital PCR hits its stride. Nat Methods.

[174] Napier MD, Haugland R, Poole C, Dufour AP, Stewart JR, Weber DJ (2017). Exposure to human-associated fecal indicators and self-reported illness among swimmers at recreational beaches: a cohort study. Environ Health.

[175] Federigi I, Verani M, Donzelli G, Cioni L, Carducci A (2019). The application of quantitative microbial risk assessment to natural recreational waters: a review. Mar Pollut Bull.

[176] Ahmed W, Hamilton KA, Lobos A, Hughes B, Staley C, Sadowsky MJ (2018). Quantitative microbial risk assessment of microbial source tracking markers in recreational water contaminated with fresh untreated and secondary treated sewage. Environ Int.

[177] García-Aljaro C, Blanch AR, Campos C, Jofre J, Lucena F (2019). Pathogens, faecal indicators and human-specific microbial source-tracking markers in sewage. J Appl Microbiol.

[178] Nappier SP, Hong T, Ichida A, Goldstone A, Eftim SE (2019). Occurrence of coliphage in raw wastewater and in ambient water: a meta-analysis. Water Res.

[179] Ahmed W, Hamilton K, Toze S, Cook S, Page D. A review on microbial contaminants in stormwater runoff and outfalls: potential health risks and mitigation strategies. Sci Total Environ. 2019. 10.1016/j.scitotenv.2019.07.055. 10.1016/j.scitotenv.2019.07.055 PMC7126443 31539962

[180] Eftim SE, Hong T, Soller J, Boehm A, Warren I, Ichida A (2017). Occurrence of norovirus in raw sewage – a systematic literature review and meta-analysis. Water Res.

[181] Kitajima M, Haramoto E, Iker BC, Gerba CP (2014). Occurrence of Cryptosporidium, Giardia, and Cyclospora in influent and effluent water at wastewater treatment plants in Arizona. Sci Total Environ.

[182] Ahmed W, O’Dea C, Masters N, Kuballa A, Marinoni O, Katouli M (2019). Marker genes of fecal indicator bacteria and potential pathogens in animal feces in subtropical catchments. Sci Total Environ.

[183] Korajkic A, McMinn BR, Ashbolt NJ, Sivaganesan M, Harwood VJ, Shanks OC (2019). Extended persistence of general and cattle-associated fecal indicators in marine and freshwater environment. Sci Total Environ.

[184] Hatvani IG, Kirschner AKT, Farnleitner AH, Tanos P, Herzig A (2018). Hotspots and main drivers of fecal pollution in Neusiedler See, a large shallow lake in Central Europe. Environ Sci Pollut Res.

[185] Neill AJ, Tetzlaff D, Strachan NJC, Hough RL, Avery LM, Watson H (2018). Using spatial-stream-network models and long-term data to understand and predict dynamics of faecal contamination in a mixed land-use catchment. Sci Total Environ.

[186] Buer A-L, Gyraite G, Wegener P, Lange X, Katarzyte M, Hauk G (2018). Long term development of bathing water quality at the German Baltic coast: spatial patterns, problems and model simulations. Mar Pollut Bull.

[187] Whitman RL, Nevers MB (2004). Escherichia coli sampling reliability at a frequently closed Chicago beach: monitoring and management implications. Environ Sci Technol.

[188] Barreras H, Kelly EA, Kumar N, Solo-Gabriele HM (2019). Assessment of local and regional strategies to control bacteria levels at beaches with consideration of impacts from climate change. Mar Pollut Bull.

[189] Kelly EA, Feng Z, Gidley ML, Sinigalliano CD, Kumar N, Donahue AG (2018). Effect of beach management policies on recreational water quality. J Environ Manag.

[190] Converse RR, Kinzelman JL, Sams EA, Hudgens E, Dufour AP, Ryu H (2012). Dramatic improvements in beach water quality following gull removal. Environ Sci Technol.

[191] Naser AM, Doza S, Rahman M, Ahmed KM, Gazi MS, Alam GR (2019). Sand barriers around latrine pits reduce fecal bacterial leaching into shallow groundwater: a randomized controlled trial in coastal Bangladesh. Environ Sci Technol.

[192] Ercumen A, Pickering AJ, Kwong LH, Mertens A, Arnold BF, Benjamin-Chung J (2018). Do sanitation improvements reduce fecal contamination of water, hands, food, soil, and flies? Evidence from a cluster-randomized controlled trial in rural Bangladesh. Environ Sci Technol.

[193] Ercumen A, Mertens A, Arnold BF, Benjamin-Chung J, Hubbard AE, Ahmed MA (2018). Effects of single and combined water, sanitation and handwashing interventions on fecal contamination in the domestic environment: a cluster-randomized controlled trial in rural Bangladesh. Environ Sci Technol.

[194] Goddard FGB, Chang HH, Clasen TF, Sarnat JA (2020). Exposure measurement error and the characterization of child exposure to fecal contamination in drinking water. npj Clean Water.

[195] Holcomb DA, Messier KP, Serre ML, Rowny JG, Stewart JR (2018). Geostatistical prediction of microbial water quality throughout a stream network using meteorology, land cover, and spatiotemporal autocorrelation. Environ Sci Technol.

[196] Capone D, Adriano Z, Berendes D, Cumming O, Dreibelbis R, Holcomb DA (2019). A localized sanitation status index as a proxy for fecal contamination in urban Maputo, Mozambique. PLoS One.

[197] Rabinovici SJM, Bernknopf RL, Wein AM, Coursey DL, Whitman RL (2004). Economic and health risk trade-offs of swim closures at a Lake Michigan beach. Environ Sci Technol.

[198] Griffith JF, Weisberg SB (2011). Challenges in implementing new technology for beach water quality monitoring: lessons from a California demonstration project. Mar Technol Soc J.

[199] Shrestha A, Dorevitch S (2019). Evaluation of rapid qPCR method for quantification of E. coli at non-point source impacted Lake Michigan beaches. Water Res.

[200] Brooks W, Corsi S, Fienen M, Carvin R (2016). Predicting recreational water quality advisories: a comparison of statistical methods. Environ Model Softw.

[201] Searcy RT, Taggart M, Gold M, Boehm AB (2018). Implementation of an automated beach water quality nowcast system at ten California oceanic beaches. J Environ Manag.

[202] Arnold BF, Schiff KC, Ercumen A, Benjamin-Chung J, Steele JA, Griffith JF (2017). Acute illness among surfers after exposure to seawater in dry- and wet-weather conditions. Am J Epidemiol.

[203] Jennings WC, Chern EC, O’Donohue D, Kellogg MG, Boehm AB (2018). Frequent detection of a human fecal indicator in the urban ocean: environmental drivers and covariation with enterococci. Environ Sci Process Impacts.

[204] Panidhapu A, Li Z, Aliashrafi A, Peleato NM. Integration of weather conditions for predicting microbial water quality using Bayesian belief networks. Water Res. 2019;115349. 10.1016/j.watres.2019.115349. 10.1016/j.watres.2019.115349 31830650

